# A Multi-Label Based Physical Activity Recognition via Cascade Classifier

**DOI:** 10.3390/s23052593

**Published:** 2023-02-26

**Authors:** Lingfei Mo, Yaojie Zhu, Lujie Zeng

**Affiliations:** School of Instrument Science and Engineering, Southeast University, Nanjing 210096, China

**Keywords:** human activity recognition, machine learning, cascade classifier, wearable devices

## Abstract

Physical activity recognition is a field that infers human activities used in machine learning techniques through wearable devices and embedded inertial sensors of smartphones. It has gained much research significance and promising prospects in the fields of medical rehabilitation and fitness management. Generally, datasets with different wearable sensors and activity labels are used to train machine learning models, and most research has achieved satisfactory performance for these datasets. However, most of the methods are incapable of recognizing the complex physical activity of free living. To address the issue, we propose a cascade classifier structure for sensor-based physical activity recognition from a multi-dimensional perspective, with two types of labels that work together to represent an exact type of activity. This approach employed the cascade classifier structure based on a multi-label system (Cascade Classifier on Multi-label, CCM). The labels reflecting the activity intensity would be classified first. Then, the data flow is divided into the corresponding activity type classifier according to the output of the pre-layer prediction. The dataset of 110 participants has been collected for the experiment on PA recognition. Compared with the typical machine learning algorithms of Random Forest (RF), Sequential Minimal Optimization (SMO) and K Nearest Neighbors (KNN), the proposed method greatly improves the overall recognition accuracy of ten physical activities. The results show that the RF-CCM classifier has achieved 93.94% higher accuracy than the 87.93% obtained from the non-CCM system, which could obtain better generalization performance. The comparison results reveal that the novel CCM system proposed is more effective and stable in physical activity recognition than the conventional classification methods.

## 1. Introduction

Physical Activity (PA), which is defined as any bodily movement produced by the skeletal muscle that results in energy expenditure [1], generally covers walking, running, cycling, sports exercise, etc. The World Health Organization (WHO) has emphasized that residents below 65 years should spend 75 min in vigorous activities and double minutes in moderate ones at least every week. Indubitably, human activity has played a crucial role in maintaining body health in daily life. Scientific and regular PA can enhance body quality and decrease the risk of getting chronic diseases, such as diabetes, dyslipidemia, and hypertension [2]. Human Activity Recognition (HAR) aims to classify the categories of skeletal muscle conducting and capture the physiological data timely through pervasive computing, which provides more precise assistance to make a remarkable contribution not only to medical diagnosis but also to the human activity research fields [3,4].

Recently, researchers have acquired information about human behavior analysis by utilizing portable mobile terminals, such as fitness trackers, smartphones, and smartwatches which have integrated a variety of inertial sensors [5]. Due to the flourishing of the Micro Electro Mechanical System (MEMS) sensor and low-power wireless technologies, PA can be measured objectively by wearable devices, which presents great advantages and feasibility. In addition to various kinds of wearable sensors, activity recognition using visual sensors has also been studied by many scholars. In this paper [6], the authors proposed a hybrid model that combines Convolutional Neural Network (CNN) and Long Short-Term Memory (LSTM) to recognize human activity using Microsoft’s motion Kinect sensor. CNN is used for spatial feature extraction, and LSTM is used to learn temporal features. However, there are some privacy problems. In the literature [7,8], the SVM, RF and Bagged DT classifiers are used to recognize the activity data collected by wearable wrist sensors. Freedson et al. [9,10] indicated the relationship between motion strength and different types using regression methods and studied the measurement of human PA by using a neural network algorithm from raw time-series signals acquired through a single accelerometer. The inertial sensors embedded in the smartphone were applied in the deep belief network to realize activity recognition, while a robust frame was established through further possessed by a kernel principal component analysis and linear discriminant analysis on the feature set [11]. It is tough to identify the PA patterns of different people who have a big range of PA behaviors. However, a sole sensor cannot reflect the physiological information completely. The multi-sensor system gauges the movements of the different body nodes, and it shows its potential to achieve promising performance in PA pattern identification. For instance, free-walking at a certain speed may bring about an acceleration that is similar to that of the same pace as holding a load, although the energy expenditure is much different. To address this drawback, some works attach attention to the combination of different kinds of sensors and then make data fused for PA recognition [12,13]. Meanwhile, ensemble learning has been increasingly investigated in the pattern recognition field. By combing the decisions of multiple classifiers or multiple sensors, the accuracy can be improved effectively [14]. For example, to capture the learning process of bipedal robot locomotion [15], a deep learning-based ensemble classifier is introduced for human lower activities recognition. Ref. [16] indicates that the ensemble of classifiers reached an agreement for activity recognition. Liu et al. [17] has realized the PA measurement precisely by multiple accelerometers and an abdominal breath sensor. Moreover, selecting the most effective component classifiers by pruning criteria was proposed to optimize the multi-sensor ensemble algorithm [18]. These distinguished studies light up and prompt many new areas of intelligent adhibition, such as healthcare monitoring, lifelogging, and fitness tracking, that use the data obtained to evaluate people’s living style and physical status.

With the rapid development of deep learning technology and its powerful ability, more and more deep learning models have been applied to the field of human motion recognition and achieved performance results. An integrated learning algorithm (ELA) based on Convolutional Neural Network (CNN) and Gated Recurrent Unit (GRU) model is proposed to recognize the activity data of smartphone sensors [19]. The literature [20] shows that personalization is more effective than deep learning in the application of traditional machine learning technology. The main objective of this paper [21] is to use a 1D convolutional neural network (1D CNN) to create a system to recognize simple everyday actions. A deep learning human activity recognition model based on residual block and Bi-directional Long Short-Term Memory (BiLSTM) is proposed [22].

In reality, an instance possessing more than one label with a high probability is ubiquitous (e.g., a movie can be regarded as both an action movie and a romance one). Hence, a strategy for multi-label has attracted a large amount of attention. The methods attached to the multi-label are grouped into some branches, such as binary relevance, label power set, classifier chain, and pruned problem transformation. Binary relevance transforms the multi-label into a series of separate binary classifications with neglecting the relevancy among the label set, which presents intuition and efficiency in the low-density label dataset [23]. In addition, the classifier chain that connects basic classifiers to guarantee the pre-label as an input of the next one is proposed to solve the independence among multiple labels [24]. While the label power set treats different sets of labels that are in a multi-label training set as a new single-label class with multi-value [25]. In contrast, with the number of new labels increasing, there would be a label-set explosion that undoubtedly enlarges the amount of computation. Ref. [26] have established a framework that combines hand gesture labels and postural activities into a multi-label activity representation to predict postural activities. The literature [27] has designed four experiments with different multi-label algorithms on activity recognition databases and points that significantly better performance is achieved by random forest with binary relevance. Physical activity can be described as not only an exact activity type but some kind according to the degree of activity intensity, that is to say, playing basketball or tennis, which are two different activities, both belong to the vigorous one as well.

In this paper, we proposed and evaluated a cascade system that adopted the cascade classifiers to establish the recognition framework with multi-label oriented (CCM). Construct a cascaded classifier to process the activity intensity and activity type label of the data instance. Firstly, the first-level base classifier mainly focuses on the characteristics of the respiratory sensor in the human activity instance and performs a predictive classification of activity intensity. Further, according to the predicted activity intensity, the second-level classifier of the corresponding intensity is selected to realize the activity class model identification. Finally, output the final prediction results and evaluate the performance of the cascade model. Expert guidance and suggestions can be provided to users to enhance their health status and fitness according to the assessment results.

To sum up, the following are the novelty and contribution.

1. We propose a cascade classifier structure for sensor-based physical activity recognition from a multi-dimensional perspective, with two types of labels that work together to represent an exact type of activity.

2. The Multi-sensor Inertial Measurement Union (IMU) has been designed and established to collect physical data and put them into storage. For the integrity and validity of the collected data, IMU has relatively arranged three sensor units on the abdomen, upper and lower limbs.

3. The aim is to use the evaluation results of the cascading model to provide expert guidance and suggestions for users to improve their health status and physique.

The remainder of the review is arranged as follows: Section 2 presents the materials and methods; the proposed method is described in detail. In Section 3, the paper is validated, and the results are discussed. Discusses the paper in Section 4. Finally, Section 5 is the conclusion of the paper.

## 2. Materials and Methods

### 2.1. Framework

Although numerous studies have focused on PA recognition, none of the works have considered the progressive relationship existing among the multiple labels. Generally, an object would be judged and divided into an extensive category by its shape, color, or other attributes, and then confirmed what it was based on the specific information. For instance, some attributes were listed, such as four wheels and running on the ground; vehicles can be derived easily. However, more details were needed to attach to determine the definite one, e.g., cars, trucks, buses, or SUVs. In this study, such a structure was proposed to apply to the area of PA recognition. Forasmuch, differing from rather utilizing a single classifier that might make the model over complex or assembling multiple classifiers with weighted majority voting. The cascade classifiers, with the help of multi-label, were designed for activity pattern recognition, which would decrease the time consumption of computation and simplify the model complexity.

**Figure 1:**
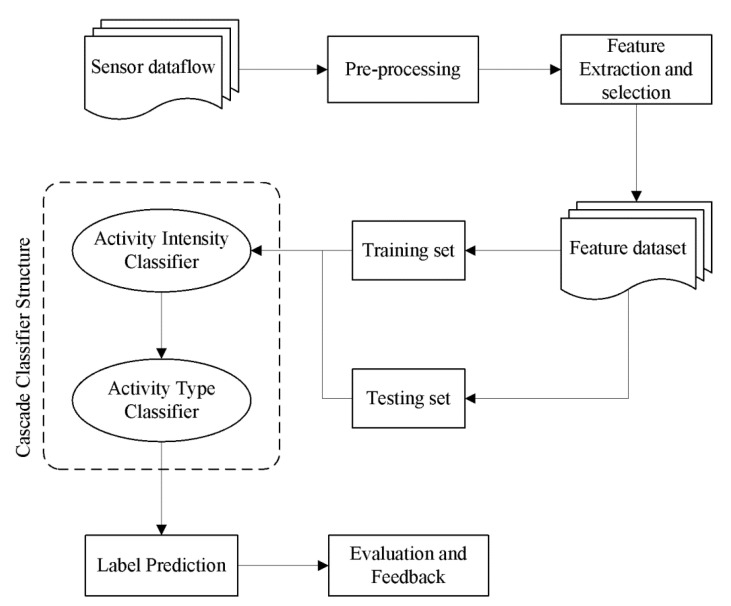
Data flow chart of the multi-label cascade classifier.

The cascade classifier structure has mainly been employed in image pattern recognition, especially in the fields of remote-sensing images, pedestrian detection, and face detection. The temporal correlation among different images obtained at the continuous moment is illustrated under the architecture of the cascade classifier for land-cover maps [28]. Tian et al. [29] has embedded weighted linear regression into a cascade structure with Haar-like features and Shapelet features to obtain outstanding pedestrian detection in the relatively complex background situation. Apart from applying in image processing, a cascade classifier structure is implemented to recognize based on the feature set from a one-dimensional data stream. A hybrid cascade model [30] has been imported to address fault detection and prediction for Android smartphones. Furthermore, the cascade system [31] also exhibits a brilliant result for each finer classification of radar signals. Based on the quoted literature, the cascade classifier structure is applied to PA recognition. The overall architecture of the PA recognition system is described in Figure 1.

By attaching multiple sensors to three nodes separately, PA signals corresponding to different joints of the human body can be obtained. The feature extraction and selection would then be performed. Given the reliable machine learning algorithm under the framework of cascade classifier, the physical activity patterns are identified, and the body performance of the individual is appraised.

### 2.2. Subjects and Materials

#### 2.2.1. Subjects

The dataset of 110 participants (including 59 females and 51 males) has been collected for the experiment on PA recognition, and all the individual characteristics are shown in Table 1. Ten PAs would be monitored for each subject, and the sensor data during the different PA patterns would be collected by the wearable measurement system correspondingly as well. Moreover, each PA pattern was performed for 5-min lasting, and then a 5-min rest period was given to adjust breathing. Before the beginning of the test, participants are allowed to lie down for reposing for about 10 min to keep the resting metabolic rate in a relatively slow and stable range. To make sure the rigorousness of the experiment, all the tests were executed during the daytime, and the participants were asked to ingest nothing except water before the data was acquired. Moreover, the whole duration of the experiment session lasts about 2 h for each individual.

For this exploratory study, a cascade classifier has proposed to attach a solution to the PA recognition based on multi-label. Note that every PA instance has included two distinct kinds of labels, one for activity intensity category and one for activity type. All ten PA patterns are divided into four categories due to the intensity or energy expenditure, as listed in Table 2.

#### 2.2.2. System Design and Realization

To reduce the disturbance of daily life due to the wearable measurement device, the inertial measurement system has equipped with some feasible functions, such as low burden and wireless connection [32]. Due to the research on the convenience of the wearable sensors, sensor positions of this system were selected; that is, the two accelerometers were placed on the wrist and hip, respectively, while the ventilation one was tied around the abdomen [33]. Hence, the Multi-sensor Inertial Measurement Union (IMU) has been designed and established to collect physical data and put them into storage. For the integrity and validity of the collected data, IMU has relatively arranged three sensor units on the abdomen, upper and lower limbs. More specifically, three sensor-node units below were involved in IMU:

1. Hip Unit: a tri-axial accelerometer ADXL345 was placed at the hip joint, which represented the degree of the lower part of the body.
2. Wrist Unit: a tri-axial accelerometer ADXL345 was placed at the wrist joint using a wristwatch-style strap which measured the physical activity signal of the upper of the body.
3. Abdomen Unit: a ventilation sensor made of piezoelectric crystals tied around the abdomen using an elastic belt was used to measure the expansion and contraction resulting from the respiration (breath rate and strength).

**Figure 2:**
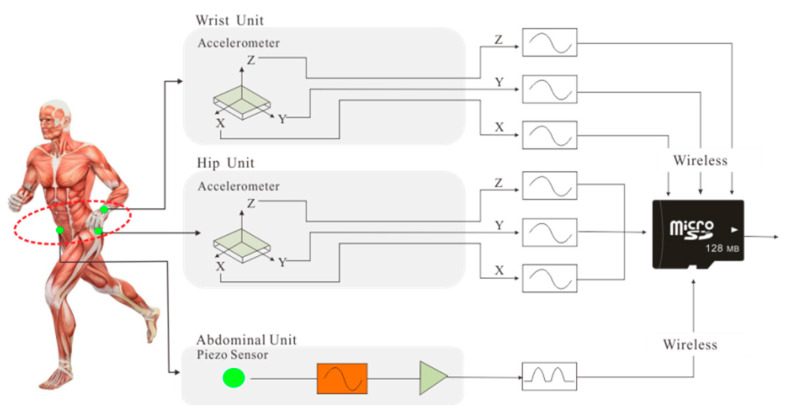
The overall architecture of the IMU system.

Figure 2 shows the architecture of the IMU system measuring the body motion parameters and respiration intensity of a human subject. The obtained data from different locations have been subsequently fused and processed to predict what the PA pattern was and quantify the energy consumption. All the data stream from these three sensor units is stored in a micro secure digital (SD) card embedded in the Hip unit.

**Figure 3:**
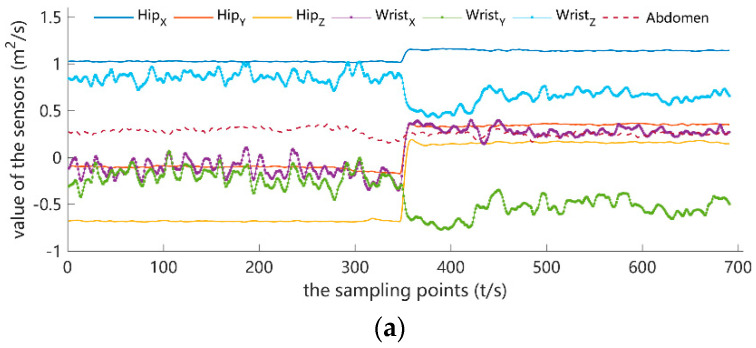

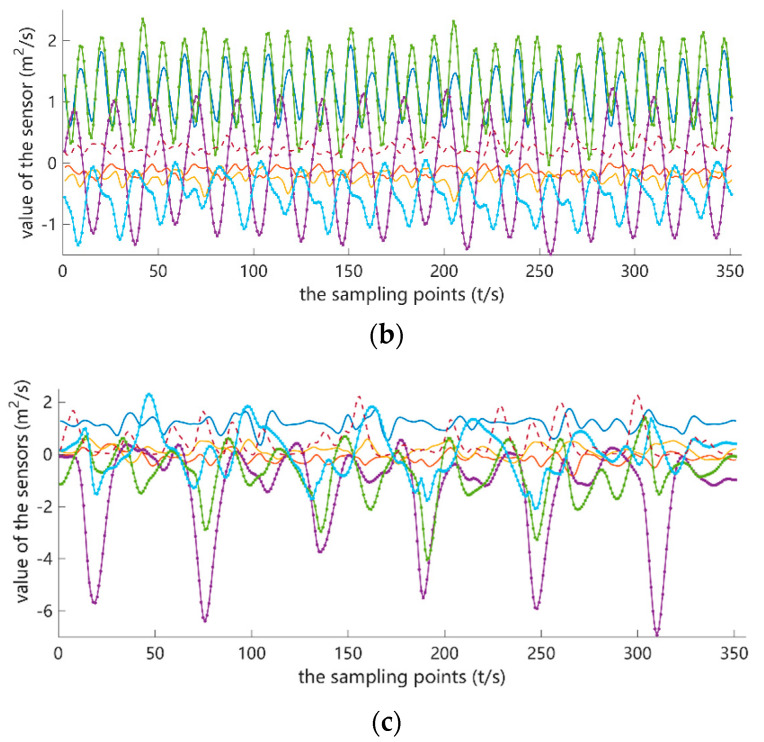
The sensors data flow wave charts of the IMU obtained, (**a**) the wave chart of the sedentary activity; (**b**) the wave chart of the TM6.0; (**c**) the wave chart of the basketball.

The acquired data stream from the IMU has been plotted in Figure 3. Among these wave charts of different PAs, there existed significant divergence according to the three measurement nodes. For example, the waveform from the hip node stayed at a more stable level than that from the wrist when the sedentary activities were performed. Because the torso of subjects maintained sitting, standing, or a stable status, upper limbs dominated the high frequency of use. As a result, the data stream from the wrist unit reflected the more detailed vibration information, which illustrated the feasibility of multi-sensor fusion to realize PA recognition to some degree. Meanwhile, owing to the more energy expenditure, playing basketball showed a higher frequency waveform than the TM 6.0’s from Figure 3b,c. Note that the differences between different PA wave charts can be the basic evidence and support to distinguish the PA pattern.

### 2.3. Signal Preprocessing

**Figure 4:**
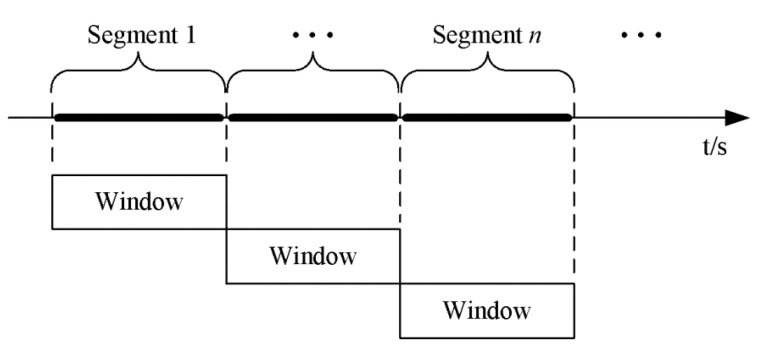
Data segmentation conducts by sliding window.

The task of data processing is divided into two main steps. The first step is time-series segmentation. Segmentation algorithms divide continuous data streams into discrete time intervals of the type expected by the information processing step [34,35,36]. The main purpose of data segmentation here is to separate the preprocessed data stream into the data segments that contain the information of complete behavior, and then the separated data segment is mainly used for the identification of feature extraction in the next step. The basic approach to this problem is to use a sliding window with a fixed length and split each time series into equal segments. Each data segment is identified by a start symbol and an end symbol that turns out to be another start symbol of the following segment at the same time. However, as the boundaries among physical activities are extremely vague, it is very difficult to split the valid sensor data stream effectively. The question that can arise here is how the recognition accuracy depends on the window length. Generally, the window size ranging from 2 s to 6.7 s is picked up among the majority of works, while a longer window length is also selected, such as 10 s and 12.8 s [4,34,35,36,37,38]. Each segment has a multi-dimensional (feature) vector extracted from it, which will be used for classification [4,39]. In this paper, a simple sliding window with no overlap was chosen for signal segmentation in Figure 4.

**Figure 5:**
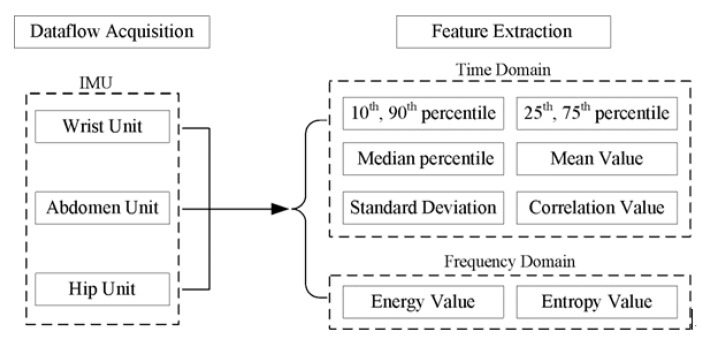
Description of the recognition feature extracted.

The other step is feature extraction and selection. Overall, Multi-domain features, including 64 features (50 time-domain and 14 frequency-domain features), were extracted for training classifiers, as shown in Figure 5. Note that the attributes of the 10th, 25th, median, 75th, and 90th percentiles represented an estimate of signal distributions in each signal. The attributes of mean and standard deviation were extracted to provide a general description of PA intensity degree. In addition, the correlation coefficient feature between the hip unit and the wrist unit was selected as well, which reflected a measurement of the coordination or variation between the upper limb and the body during an activity. Frequency-domain features (energy and entropy) have been extracted separately for these two accelerometers. As for the ventilation sensor on the abdomen, the breathing frequency was decided by the dominant frequency of the respiratory signal obtained from a spectral analysis. Meanwhile, to avoid the situation that the features in the smaller numeric ranges could be overwhelmed by those of greater numeric values, normalization was necessarily applied to convert the extracted features into the range from 0 to 1 [40].

Based on the dataset of the human body, the accelerometer of the hip and wrist and the data of the respiratory telescopic sensor of the abdomen were selected in this paper for time domain characteristics and frequency domain characteristics.

Mean is the average level of signal values in the index frame, which can be calculated by the formula:

(1) $$Mean=\frac{1}{N}\sum_{n=1}^{N}X_{n}$$

where $X_{n}$ indicates the sensor sequence and N indicates the sequence length.

Variance (VR) describes the degree of data dispersion of a signal around the arithmetic mean. The formula:

(2) $$VR=\sigma^{2}=\frac{1}{N}\sum_{n=1}^{N}\left(X_{n}-\bar{X}\right)$$

where σ is the data of standard deviation, $\bar{X}$ is the average sensors data.

The Correlation Coefficient (*CC*) considers the degree of correlation between data at different locations. For two signal sequences X and Y, the correlation coefficient between them can be expressed as:

(3) $$CC_{\left(X,Y\right)}=\frac{\mathrm{cov}\left(X,Y\right)}{\sigma_{X}\times \sigma_{Y}}$$

where $\mathrm{cov}\left(X,Y\right)$ represents the covariance of the two, $\sigma_{X}$ and $\sigma_{Y}$ represent their standard deviations, respectively.

Energy is the average power of the signal $X_{n}$ over the time interval (−*N*/2, *N*/2). The signal spectrum is obtained by the fast Fourier transform, and the power signal of the spectrum is the sum of the squares of the spectrum modes, so the energy can be calculated by the following formula:

(4) $$E_{\left(X\right)}=\frac{1}{N}\sum_{n=1}^{N}{\left|F,\left(e^{jw}\right)\right|}^{2}$$

where $F\left(e^{jw}\right)$ is the amplitude of the $X_{n}$ fast Fourier transform.

Spectral Entropy (SE) is the subframe entropy of normalized spectral energy. To calculate the spectral entropy of the frame i, each signal frame is first divided into K subframes of fixed size. Then, the spectral energy of each subframe is calculated and divided by the total spectral energy of the signal frame. The spectrum entropy formula is:

(5) $$H_{i}=-\sum_{k-1}^{K}n_{k}\log_{2}\left(n_{k}\right)$$

where $n_{k}=\frac{E_{k}}{\sum_{j=1}^{K}E_{j}}E_{k}$ is the spectral energy of the subframe.

To map the original activity data to different category Spaces, it is necessary to analyze its statistical characteristics, such as mathematical distribution and extract the recognition feature vectors that can represent different human activities from different dimensions. However, too many feature vectors will bring some irrelevant or redundant information, which will affect the accuracy of tag prediction. In this paper, 49 time-domain features, 1 correlation feature and 14 frequency-domain features have been extracted for the following pattern recognition. To realize the diversity of the training of each base classifier, 70% of the overall features were picked up randomly for the training of the classifier.

### 2.4. Machine Learning Model

Machine Learning uses algorithms to analyze existing data to acquire knowledge and then apply it to new data. In this paper, three machine learning algorithms are used to train the model. Including Random Forest (RF), Sequential Minimal Optimization (SMO) and K Nearest Neighbors (KNN).

Random Forest (RF) was proposed by Breiman in 2001. As a general classification and regression method, it combines several random decision trees and shows excellent performance in an environment where the number of variables is much larger than the number of observations through the average fusion mechanism. Based on the simple and feasible voting mechanism of random Forest and its high and stable accuracy, random Forest has been widely used in medicine, text classification and facial recognition.

A Support Vector Machine (SVM) classifier is a supervised learning algorithm based on statistical theory. It is mainly used in the fields of regression analysis and pattern recognition. It can minimize the empirical errors of data while maximizing geometric edges, providing excellent generalization performance. Based on the SVM algorithm, Shevade et al. proposed an iterative algorithm of Sequential Minimal Optimization (SMO), which can effectively replace vacancy values in data and can effectively solve multi-class classification problems by using kernel functions of Gaussian kernel.

k-Nearest Neighbor (kNN), unlike Eager Learning algorithms such as random forest, needs to learn a model on the training sample set according to certain rules or algorithms and then classify test samples. The Negative Learning algorithm (Lazy Learning) represented by kNN is to jointly model test samples and training samples.

### 2.5. Recognition Module

As an open-source data mining platform, Waikato Environment for Knowledge Analysis (WEKA) brings together a large number of machine learning algorithms that can undertake data mining tasks through visualization on a new interactive interface. The whole PA recognition frame is architected by Java, relying on the WEKA toolkit.

A two-layer cascade classifier structure is adopted to build the classification system (Algorithm 1). To avoid similar data from the same participant appearing in the testing set, make sure the irrelevance between the testing set and training set when every iteration. Note that the pre-layer aims to classify the intensity category label by selecting some features and then assigning the corresponding classifier according to the outcome from the pre-one in the second layer. Ultimately, the final prediction will be given through the cascade architecture.
**Algorithm 1:** The pseudo-code of cascade classifier based on multi-label (CCM) algorithm.**Inputs:** **Instances**, a sequence of *n* instances {(x_1_,y_1_,y_1_′), …, (x_n_,y_n_,y_n_′)} with two kind of labels, y_i_, y_i_′ ϵ *Y* = {1, …, *k*}. **SubjectSet**, a collection of key values of all the participants. **1: foreach** *sub.Id* **in** SubjectSet: **2:           iterate** *instance* **in** Instances: **3:         if** *instance*.subId = *sub.Id* **then**: **4:            ** put *instance* into the testing set **5:         else if** **6:            ** put *instance* into the training set **7:           end iterate** **8:          ** build the activity intensity model_Layer1 (training set) **9:          ** build the activity type models_Layer2 (training set) **10:        ** validate the Model_Layer1 (testing set) **11:        ** get the label of layer1 **then**: **12:       ** validate the models_Layer2 (testing set) **13:       ** activity label ← get the label of the layer2 **14:          return** activity label **15: end for** **16: output:** activity label

## 3. Experiments and Results

### 3.1. Performance Metrics

To better evaluate the performance of the classifier, some performance metrics are adopted, such as accuracy, sensitivity, specificity, precision, and F1-score. The performance measures used are described below.

(6) $$\mathrm{Accuracy}=\frac{TP+TN}{TP+FP+TN+FN}$$

where TP denotes the true positive of the elements, TN denotes true negative, FP indicates the false positive, and FN indicates the false negative.

(7) $$\mathrm{Sensitivity}=\frac{TP}{TP+FN}$$

(8) $$\mathrm{Specificity}=\frac{TN}{TN+FP}$$

(9) $$\mathrm{Precision}=\frac{TP}{TP+FP}$$

(10) $$F1\text{-}\mathrm{score}=\frac{2\cdot \mathrm{Sensitivity}\cdot \mathrm{Precision}}{\mathrm{Precision}+\mathrm{Sensitivity}}$$

### 3.2. Experiments

To validate the feasibility of this proposal, the leave one individual out cross-validation has been adopted to split the training set and the testing set, differing from the ordinary cross-validation. That is to say, the data of every participant need to be the testing set, and the rest are put into the model to configure the model’s set parameters. This validation can effectively avoid the repeatability of instances sampled from the same participant to guarantee the irrelevance between the training set and testing set, which decreases the over-fitting and makes prediction more acceptable. However, if all the participants have been iterated to be the testing set, it would take on much time expenditure. As a result, the time consumption would be taken into consideration as well. To balance the experiment’s feasibility and time expenditure, 60 of the 110 participants are selected randomly to constitute the testing set.

**Figure 6:**
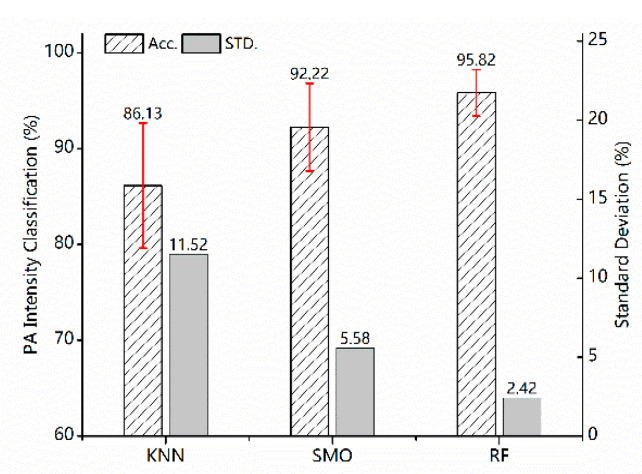
Classification results aimed at the four activity intensity labels among these three machine learning algorithms.

Based on that, the selected testing sets have been marked with the intensity label through three machine learning algorithms. The pre-layer performance-validated experiments have been conducted with 60 loops, and the result of PA classification is presented in Figure 6 below. Table 3, Table 4 and Table 5 show the Confusion matrix of the three algorithms for the four activity intensity labels. Performance metrics result aiming at the four activity intensity labels among these three machine learning algorithms in Table 6.

**Figure 7:**
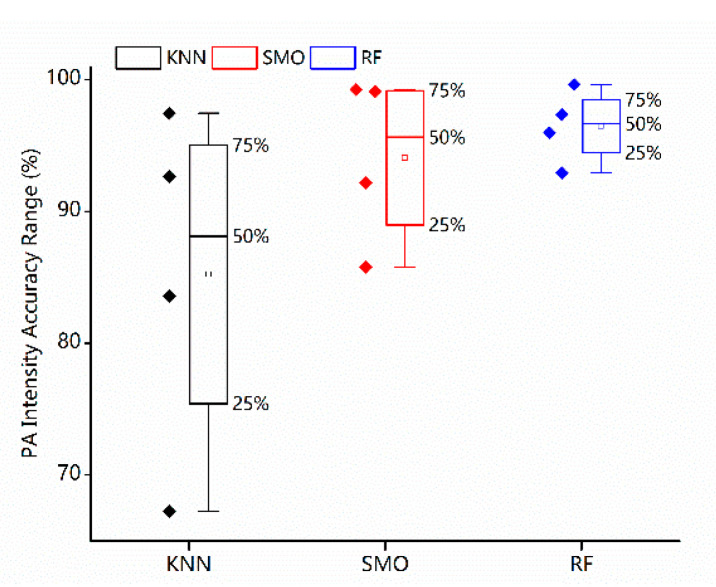
Statistical distributions of the first layer classification results.

The bar chart shows that all the methods have been equipped with strong classification capabilities aiming at the four target classes (sedentary, household, moderate and vigorous. Especially the RF has achieved the best performance, and the mean classification accuracy reaches up to 95.82%. Meanwhile, the standard deviation low at 2.42%. SMO and KNN have yielded the mean classification accuracy of 92.22% and 86.13% separately, with a standard deviation of 5.58% and 11.51% as well, which reveals the balanced and outstanding classification effects for the four intensity labels. In addition, the box diagram of the first layer classification results is also shown in Figure 7. As for these four intensity categories, all three classifiers have different classification accuracies. Note that the RF has reflected the balanced performance at a higher level.

**Figure 8:**
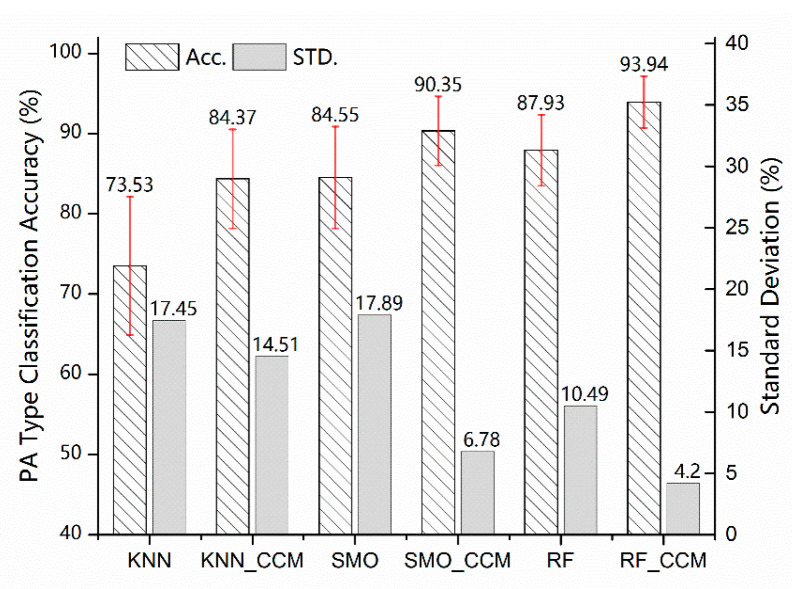
The performance comparison between the CCM and non-CCM classifiers.

After the computing of the first classification layer, each instance has been divided into the type of PA intensity which belongs to. According to the first layer prediction result, the corresponding classifier in the second cascade layer would be assigned to finish the more detailed PA recognition task. Through the structure of the cascade classifier, every base classifier just needs to attach attention to the minority PA patterns within the specific activity intensity label, which can simplify the complexity of the model and reduce the amount of calculation. As mentioned before, the testing set and the training set are separated in the second layer with the same method in the pre-layer, and leaving one individual out cross-validation is taken to verify the performance of the whole recognition cascade structure as well. When an individual comes to be the testing set, the training set of the classifiers in the second layer would not involve the data from that individual either. Based on that, the whole system performance-validated experiments have been carried out. To illustrate the improvement of the cascade classifier based on the multi-label that this study proposes, some experiments which directly use the three machine learning algorithms to realize the PA pattern recognition have been conducted for comparison, and the results of the PA classification are presented in Figure 8 below. The performance metrics used to evaluate the proposed method are in Table 7.

**Figure 9:**
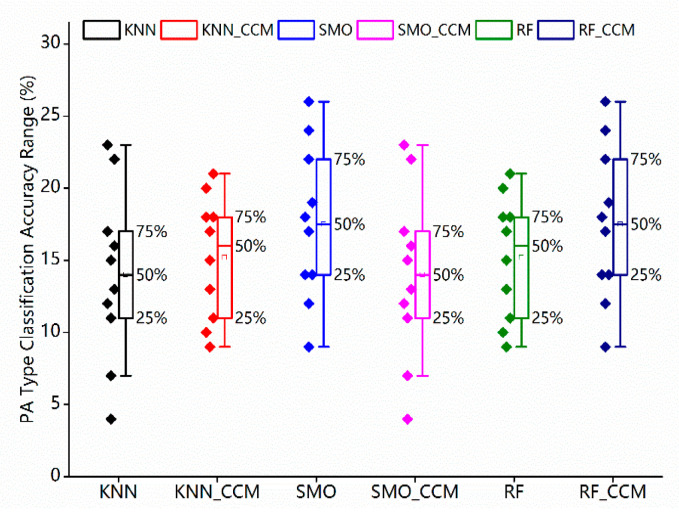
Statistical distributions of classification results among the equipped with CCM structure algorithm and the normal classification ones.

Figure 8 shows that the multi-label cascade classifier architecture has all performed better in classification than the normal one for these 10 PA patterns. There is a significant trend among all three selected algorithms; that is, the improvement of the PA pattern recognition accuracy highlighted after importing the multi-label cascade structure. Compared with normal direct classifying, the cascade classifier approach based on the RF enhances the mean accuracy from 87.93% to 93.94% with about a 6.8% rise and decreases the standard deviation from 10.49% to 4.20% sharply. The same for the KNN and SMO, the mean classification accuracies both achieve improvement which is from 73.53% and 84.55% to 84.37% and 90.35%, separately and the standard deviations lower to 14.51% and 6.78%, respectively. In addition, the statistical distributions of classification results of the validation experiment are clustered in Figure 9. For the non-CCM classifier, all the data obtained from the multiple sensors have been merged as one single set of features. The non-CCM classifier has to handle all the PA patterns. Taking the RF algorithm, for example, the confusion matrices of the classification accuracies of the 10 different PA patterns of the cascade classifier and non-cascade classifier are listed in Table 8.

Comparing the confusion matrices in Table 8, the classification accuracy of the CCM system has improved significantly, especially for filing paper (from 76% to 95.4%) and vacuuming (from 63% to 95.8%). Otherwise, the model to classify the cycling with 1-kp resistance and the tennis pattern also shows a certain degree of improvement. Although there are some negative impacts on some PA patterns, the whole classification accuracy has achieved growth. More specifically, all three machine learning algorithms with CCM have a rational statistic distribution and a higher benchmark, which illustrates that the CCM system realizes better effectiveness for the 10 PA patterns. Note that the lower the standard deviation, the smaller the fluctuation range of the accuracy for each PA pattern, indicating that the proposed approach has obtained satisfactory performance for all labels either, except for the total accuracy.

## 4. Discussion

In this paper, a novel multi-label cascade classifier system has been proposed and adopted for daily physical activity pattern recognition and achieves a promising performance. The wearable inertial measurement device has been designed to acquire the body motions information and respiration rate, which consists of a ventilation sensor around the abdomen and two tri-axial accelerometers placed on the wrist and the hip separately. Compared with the traditional single accelerometer measurement, applying multiple inertial sensors can measure and provide more detailed information about body movements. Meanwhile, a ventilation sensor enhances the additional measurement of respiration expenditure and physical activity energy expenditure. Multi-label is addressed to make a supplement for activity pattern recognition. An object that owns more than one label is a more common phenomenon. By adding the PA intensity labels, one extra indicator would support making the instances divided into the correct category as much as possible.

Generally, the current PA recognition systems have acquired quite an acceptable classification performance by using ensemble learning (integrating the multiple different classifiers based on the classification accuracies of the different PA patterns) and reliable decision fusion strategies, such as the instance-specific weighted majority voting. However, the higher classification accuracy has been obtained at the expense of taking more computational resources than non-ensemble classifiers. Meanwhile, the complexity of the ensemble model built is too redundant, and all PA patterns need to be judged in every base classifier, which enlarges the scale of models and enhances the difficulty of classification. As a result, unnecessary time consumption is also a factor to consider undoubtedly. Based on that, a cascade classifier structure based on a multi-label (CCM) approach has been designed and evaluated for the physical activity measurement and recognition system and simplifies the classification ranges of the classifiers. In this study, the cascade classifier structure based on the multi-label (CCM) approach has been designed and evaluated for the physical activity measurement and recognition system. A two-cascade structure has been established. The first layer mainly focuses on the classification of PA intensity categories and assigns the corresponding classifier in the second layer according to the prediction of the first layer to realize the PA recognition. Comparing the classifiers with non-CCM, the CCM approach has shown better performance, that is, higher mean accuracies and lower standard deviations.

Moreover, the CCM system has demonstrated better generalization capability than the non-CCM one. As seen in the leave one individual out cross-validation results, the CCM approach has presented a better performance on the PA classification of new test subjects, while in contrast, lower classification accuracies have been obtained from the same test subjects when using the non-CCM model. The cascade classifier approach, on the other hand, maintains the statistical distribution of each sensor dataset of its own and makes each classifier devoted to the minority of PA patterns. It is seen that the classification performance is reliable and robust in generalization due to the variability among the participants being reduced significantly. In the implementation, the RF-CCM structure classifier is selected first. In the process of training, all the training datasets are used for training, and the optimal parameters of the first layer are obtained and fixed, and then the optimal parameters of the second layer are obtained. Then, the final model will be used for testing.

Despite the promising performance that has been illustrated in this study, it is notable that some shortcomings still need to be mentioned. For instance, the cascade classifier structure exists the phenomenon of error passed and superimposed; that is, an instance must be divided into a wrong category no matter how precise the classifiers are in the second layer if the first layer gives a misclassification. As a consequence, measures need to be taken to optimize the cascade structure and reduce the error passed and superimposed. On the other hand, more subjects will be involved in later research to enhance the robustness of generalization. Furthermore, several issues remain discussed as follows,

1. The selection and comparison of sliding window length, features, and base classifiers.
2. Number and placement of the wearable device are arranged to acquire a better classification performance.
3. Optimization of the CCM structure to decrease the error accumulation.

We hope that these matters will be addressed in future studies to further improve the performance and generalization capability of the multi-label-based cascade classifier system.

## 5. Conclusions

In this paper, a novel solution, a cascade classifier structure, is proposed to recognize multi-label human activities. The first-level base classifier mainly classifies the labels reflecting activity intensity, and then according to the predicted output, is data instance selects the corresponding activity type classifier to realize activity category pattern recognition. The performance of this method is verified on the self-collection database. The promising results indicate that the proposed method could be efficiently identified multi-label physical activity.

## Figures and Tables

**Table 1:** Statistic distribution and characteristics of the participants.

Categories	Statistic Information			Mean	STD
Age (years)	20–30	30	27.3%	38.71	11.83
	30–40	28	25.5%		
	40–50	25	22.7%		
	50–60	27	24.5%		
Mass (kg)	<50	2	1.8%	71.25	14.86
	50–60	26	23.7%		
	60–70	34	30.9%		
	70–80	13	11.8%		
	80–90	21	19.1%		
	>90	14	12.7%		
Height (cm)	150–160	16	14.5%	169.5	9.24
	160–170	43	39.1%		
	170–180	33	30.0%		
	>180	18	16.4%		
BMI (kg/m^2^)	<18.5	1	0.9%	25.02	4.21
	18.5–25	65	59.1%		
	25–30	30	27.3%		
	>30	14	12.7%		

**Table 2:** Physical Activity types obtained for the experiment.

Activities Type	Intensity Category	Abbr.
Computer work Filing paper	Sedentary activity	CW FP
Moving boxes Vacuuming	Household and other	MB VA
Cycling with 1-kp resistance Treadmill at 3.0 mph Treadmill at 4.0 mph	Moderate activity	C1 T3 T4
Treadmill at 6.0 mph Tennis Basketball	Vigorous activity	T6 TE BA

**Table 3:** The Confusion matrix of the four activity intensity labels among the KNN algorithm.

		Predictive Labels			
		Sedentary	Household	Moderate	Vigorous
Real Labels	Sedentary	280	27	26	1
	Household	8	275	31	2
	Moderate	40	89	843	3
	Vigorous	7	18	10	229

**Table 4:** The Confusion matrix of the four activity intensity labels among SMO algorithm.

		Predictive Labels			
		Sedentary	Household	Moderate	Vigorous
Real Labels	Sedentary	220	4	3	0
	Household	1	153	49	0
	Moderate	1	3	446	2
	Vigorous	0	6	22	261

**Table 5:** The Confusion matrix of the four activity intensity labels among RF algorithm.

		Predictive Labels			
		Sedentary	Household	Moderate	Vigorous
Real Labels	Sedentary	220	3	4	0
	Household	2	190	11	0
	Moderate	1	3	447	1
	Vigorous	3	2	19	265

**Table 6:** Performance metrics result aiming at the four activity intensity labels among these three machine learning algorithms.

Algorithms	Accuracy	Sensitivity	Specificity	Precision	F1-Score
KNN	0.8613	0.8602	0.9506	0.8523	0.8520
SMO	0.9222	0.9032	0.9698	0.9406	0.9181
RF	**0.9582**	**0.9528**	**0.9842**	**0.9646**	**0.9580**

**Table 7:** The performance metrics comparison between the CCM and non-CCM classifiers.

Algorithms	Accuracy	Sensitivity	Specificity	Precision	F1-Score
KNN	0.7353	0.7030	0.9689	0.6957	0.6993
KNN-CCM	0.8437	0.8130	0.9822	0.8263	0.8196
SMO	0.8455	0.8185	0.9720	0.8117	0.8151
SMO-CCM	0.9035	0.9075	0.9888	0.9153	0.9114
RF	0.8793	0.8807	0.9810	0.8851	0.8829
RF-CCM	**0.9394**	**0.9422**	**0.9930**	**0.9381**	**0.9401**

**Table 8:** The classification accuracies of the 10 PA patterns comparison between the RF with CCM and the RF with non-CCM (%).

Authentic Labels		Predicted Activity Labels									
		CW	FP	MB	VA	C1	T3	T4	T6	TE	BA
RF Non-CCM	CW	**97.6**	17.8	0	5.2	0.5	0.5	0.4	1.3	0	0
	FP	0.6	**76.0**	0	1.3	0	0	0	0	0	0
	MB	0	0	**88.3**	5.2	5.3	0.3	0	0	3.2	0
	VA	0	0	0	**63.0**	0	0.5	0.8	0	0	0
	C1	0.6	1.6	1.8	0	**90.7**	0	0	0	0	0
	T3	0.6	0.8	3.7	21.4	1.5	**93.1**	0.8	0	1.6	0
	T4	0	0	6.2	2.6	1.5	5.3	**93.7**	0	1.6	0
	T6	0	0	0	0	0	0	0.4	**98.7**	0	0
	TE	0.6	3.8	0	1.3	0.5	0	0	0	**83.9**	5.6
	BA	0	0	0	0	0	0.3	3.9	0	9.7	**94.4**
RF CCM	CW	**95.9**	1.3	0	0	0	1.8	0	0	0	0
	FP	2.7	**95.4**	0	2.1	0	0.6	0	0	0	0
	MB	0	0	**89.1**	0.7	0	0.6	0	0	0	0
	VA	0	1.3	5.5	**95.8**	0	3.5	1.7	0	0	0
	C1	0	0	3.6	0	**94.8**	0	0	0	0	0
	T3	1.4	0	0	0	1.3	87.0	0.4	0	0	0
	T4	0	0	1.8	0	3.9	3.5	**91.9**	0	3.7	0
	T6	0	1.3	0	0	0	0	2.6	**99.2**	3.7	0
	TE	0	0	0	0	0	0.6	0	0	**88.9**	0
	BA	0	0.7	0	1.4	0	2.4	3.4	0.8	3.7	**100**

## Data Availability

The data was collected by the Department of Kinesiology at the University of Massachusetts, supported by NIH (National Institutes of Health) of the USA under the grant UO1-CA130783.
